# A genomic perspective on South American human history

**DOI:** 10.1590/1678-4685-GMB-2022-0078

**Published:** 2022-07-29

**Authors:** Marcos Araújo Castro e Silva, Tiago Ferraz, Tábita Hünemeier

**Affiliations:** 1 Universidade de São Paulo, Instituto de Biociências, Departamento de Genética e Biologia Evolutiva, São Paulo, SP, Brazil. Universidade de São Paulo Instituto de Biociências Departamento de Genética e Biologia Evolutiva São Paulo SP Brazil

**Keywords:** Native Americans, genomics, peopling South America, population dynamics

## Abstract

It has generally been accepted that the current indigenous peoples of the Americas are derived from ancestors from northeastern Asia. The latter were believed to have spread into the American continent by the end of the Last Glacial Maximum. In this sense, a joint and in-depth study of the earliest settlement of East Asia and the Americas is required to elucidate these events accurately. The first Americans underwent an adaptation process to the Americas’ vast environmental diversity, mediated by biological and cultural evolution and niche construction, resulting in enormous cultural diversity, a wealth of domesticated species, and extensive landscape modifications. Afterward, in the Late Holocene, the advent of intensive agricultural food production systems, sedentism, and climate change significantly reshaped genetic and cultural diversity across the continent, particularly in the Andes and Amazonia. Furthermore, starting around the end of the 15th century, European colonization resulted in massive extermination of indigenous peoples and extensive admixture. Thus, the present review aims to create a comprehensive picture of the main events involved in the formation of contemporary South American indigenous populations and the dynamics responsible for shaping their genetic diversity by integrating current genetic data with evidence from archeology, linguistics and other disciplines.

## The first humans on the world’s last unexplored continent

At the end of the last ice age, the arrival of the first groups of Homo sapiens in the Americas, at least 16 ka BP (kilo-annum Before Present), marks the beginning of human history on the last continent uninhabited by hominins. These newcomers were descended from Northeast Asian peoples, as demonstrated by a vast wealth of evidence gathered over more than a century from multiple fields of science (Skoglund and Reich 2016; Potter et al., 2017; Braje et al., 2017; Waters 2019; Mendes et al., 2020; Willerslev and Meltzer, 2021). Indeed, this hypothesis was proposed very early (de Acosta 1589), due to the evident morphological similarities between Native Americans and Asians. Several lines of evidence reveal that the indigenous peoples of the Americas are descendants of migrants who crossed the Beringian continental shelf from Siberia to Alaska. This passage likely occurred around the last glacial maximum (LGM) period, which happened roughly between 26.5 to 19 ka BP, when the world’s ice sheets were at their peak and ocean levels were at their lowest point, which was around 130 meters below current levels, exposing vast swathes of land (Clark *et al.*, 2009, Lambeck et al., 2014).

**Figure 1.**
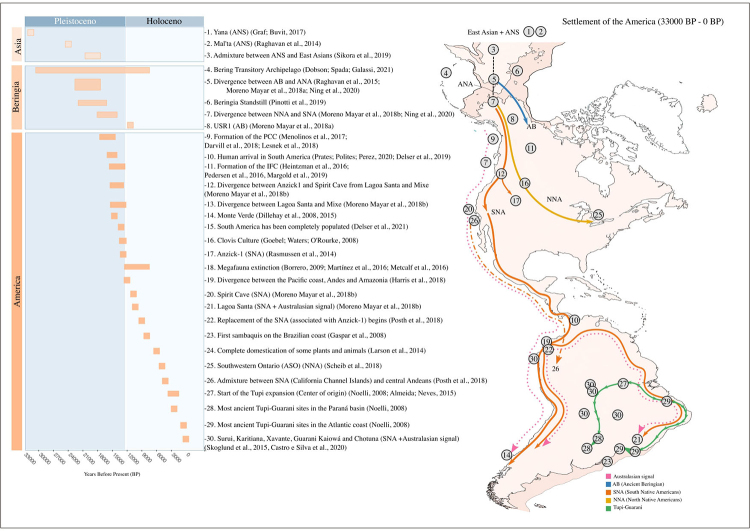
Summary of the population history of indigenous Americans. On the left is a timeline of the main milestones in human history in the Americas, which are numbered, described, and referenced. Furthermore, the panels are subdivided by continent (Asia, Beringia, and America) and geological period (Pleistocene and Holocene). The map on the right depicts the approximate positions of some of the major landmarks (points), as well as the probable routes (hypotheses) of dispersion (arrows), however these should not be interpreted literally, since they simply approximate the direction of these movements.

The settlement of the Americas likely took place after the initial influx of human populations into East and Northeast Asia; therefore, the comprehension of the peopling of America requires first a contextualization of the human dispersion in East and Northeast Asia. Northeast Asia was settled by humans before the LGM, as pointed out by the most ancient archeological evidence: the Yana River site with approximately 31,6 ka BP, near the coast of the Arctic Ocean in northeast Russia (Graf and Buvit, 2017), and the Mal’ta site with 24 ka BP, in south-central Siberia (Raghavan et al., 2014) (Figure 1). In this period, the northeastern region of Asia was occupied by a population known as the Ancient North Siberians (ANS), which diverged from the western Eurasians around 39 ka BP, shortly after their divergence from the East Asians at 43,1 ka BP (Sikora et al., 2019). The ANS exhibit a genetic affinity with both contemporary Native Americans and Northern Europeans, a pattern not seen in other Eurasians, not even in more ancient ones like those discovered in Sunghir, western Russia, with 34 ka BP (Sikora *et al.*, 2017), and in Tianyuan, southeastern China, with 39,6 ka BP (Fu et al., 2013) which have higher affinities with western Eurasians and East Asians, respectively. Although the ANS have not survived to the present day as a separate people, through an admixture event with an East Asian group approximately 20-18 ka BP, they gave rise to the ancestors of the Native Americans (ANA) and the Ancient Paleo-Siberians (Figure 1). 

As proposed by the Beringian standstill hypothesis, the ANA would have entered a period of relative isolation from other groups before or during their first dispersal to the American continent (Tamm et al., 2007; Kitchen et al., 2008; Mulligan et al., 2008). This hypothesis is mainly supported by exclusive patrilineal and matrilineal lineages (i.e., NRY and mtDNA haplotypes) in the Americas (Fagundes et al., 2018; Bergström et al., 2020; Bisso-Machado and Fagundes, 2021). This period of isolation would have lasted between 4,6K (Pinotti et al., 2019) and 15K years (Graf and Buvit, 2017), and likely took place in Beringia (Figure 1). Although there is no consensus on where and why this event occurred, some possibilities are that it was caused by the existence of ecological barriers (Tamm *et al.*, 2007) and/or that Beringia was a bioclimatic refugium during the LGM (Sikora et al., 2019; Rae et al., 2020).

The Ancient Beringians (AB) split from the ANA (Raghavan et al. 2015; Moreno-Mayar et al., 2018a) between 22-18 ka BP, after that, approximately between 17,5-14,6 ka BP, the ANA became genetically structured (Moreno-Mayar *et al.*, 2018b), giving rise to the northern Native Americans (NNA) and southern Native Americans (SNA) (Figure 1) (Scheib et al., 2018; Posth et al., 2018; Moreno-Mayar *et al.*, 2018b). Traditionally eastern Beringia was considered the place where this diversification process occurred (Potter et al., 2018; Waters, 2019); however, new lines of genetic evidence suggest that the split between AB and ANA occurred in northeast Asia and Siberia (Moreno-Mayar *et al.*, 2018a), so that the Beringian population would already be structured.

## On the way to a new hemisphere

Massive continental glaciers prevented an interior path to the American continent from Beringia during the LGM (Meltzer, 2009). Nevertheless, at the end of the LGM, an ice-free corridor (IFC) arose along the Rocky Mountains (Perego et al., 2009; Potter et al., 2018), separating the Cordilleran ice sheet to the west from the Laurentide glacier to the east (Figure 1). This IFC was an ecologically viable passage to humans only about 15-13 ka BP (Heintzman et al., 2016; Pedersen et al., 2016; Margold et al., 2019). However, alternative routes were possible, such as the scenario in which the earliest migrants arrived through a Pacific coastal corridor (PCC) at least between 17-15 ka BP, which currently stands as the most likely based on current evidence (Figure 1) (Fagundes *et al.*, 2008; Perego *et al.*, 2009; Menounos et al., 2017; Lesnek et al., 2018; Darvill et al., 2018; Delser et al., 2021). It is important to note that both pathways are not mutually exclusive, and so theoretically, both could have been used (Potter *et al.*, 2018), albeit, if so, at quite different times. 

Hitherto all indigenous Americans studied, with few exceptions, are exclusively descendants from the NNA and/or SNA branches, in turn all South Americans studied thus far have SNA ancestry, therefore the initial dispersion into South America must have happened only after the SNA and NNA branches diverged, placing an upper time limit on this event (Figure 1) (Posth et al., 2018). Some populations in Central and South America appear to have contributions from both of these lineages (Scheib et al., 2018), although this mixture likely occurred after the initial settlement. Speakers of the Na-Dené and Eskimo-Aleut languages, which live in northern North America, are also notable exceptions to this pattern of exclusive NNA/SNA ancestry because to properly explain their genetic diversity further gene flow from Asian groups is required (Rasmussen et al., 2010; Reich et al., 2012; Moreno-Mayar et al., 2018a; Flegontov et al., 2019).

Furthermore, the widespread distribution of a specific set of very characteristic projectile points along with a specific set technological artifacts first discovered in Clovis, New Mexico, in the southwestern United States (Figgins and Cook, 1927), was thought to be evidence of the first Americans, who would have been megafauna hunters, according to a long-held hypothesis, known as the Clovis first hypothesis (CFH) (Haynes, 1964). However, a growing body of evidence contradicting this hypothesis has accumulated over time, indicating that the so-called Clovis culture was relatively short-lived, with dates ranging around 13-12,7 ka BP (Figure 1) (Goebel et al., 2008), significantly later than both the timeframe when the passage into the Americas became available (17-15 ka BP) (Menounos et al., 2017; Lesnek et al., 2018; Darvill et al., 2018) and the age of most ancient archeological sites in North and South America (Gilbert et al., 2008; Dillehay, 2009; Dillehay *et al.*, 2015). The CFH is also incompatible with the divergence dates inferred for the most deeply diverged SNA lineages, first, the common ancestors of the Anzick-1 (12,8 ka BP) and Spirit Cave (10,7 ka BP) diverged around 14,9-13,2 ka BP from the ancestors of Lagoa Santa (10,4 ka BP) and of the contemporary Mixe ethnic group, and next the later ones would have diverged from each other around 14,8-12,8 ka BP (Figure 1) (Moreno-Mayar et al., 2018b). Conversely, this population history is aligned with the pre-Clovis sites dates and the inferred arrival of humans in the South American continent around 15,5-14,6 ka BP (Figure 1) (Prates et al., 2020).

It’s important to note that the archeological sites mentioned above are all post-LGM. However, several pre-LGM sites have been reported, suggesting that human arrival in America might have occurred far earlier. The Pedra Furada (Guidon, 1986) and the Santa Elina (Vialou, 2003) are two of the most notable pre-LGM sites in South America in Brazil - in Piauí, and Mato Grosso states, respectively - and exhibiting dates as early as 50 and 30 ka BP. Unlike post-LGM sites, which are widely accepted, pre-LGM sites are still a subject of debate (Sutter, 2021). Beyond that, as previously discussed, human dispersion into America necessarily occurred after the arrival of humans in Northeastern Asia and Siberia, which happened only in the last 32 ka BP (Figure 1) (Graf and Buvit, 2017). As a result, while it is theoretically possible that groups of modern humans arrived on the American continent before the LGM, archeological evidence of their presence is extremely rare, and their contribution to Native American genetic composition would be null or negligible. Indeed, genetic evidence of this early presence may have been discovered (Raghavan et al., 2015; Skoglund et al., 2015; Posth et al., 2018; Moreno-Mayar et al., 2018b; Castro e Silva et al., 2021); however, it is yet unclear whether or not this is the case, as will be explored further below.

## A changing time horizon

Considering a post-LGM initial settlement scenario, not considering controversial South American sites dating from the pre-LGM period, the dispersal of the first migrants from North to South America must have been extremely rapid. According to available evidence, the first humans in South America arrived as early as 15-14 ka BP (Bodner et al., 2012; Rasmussen et al., 2015; Dillehay et al., 2017), although one estimate based on the probability distribution of archeological site dates pushes the initial arrival to approximately 15,5 ka BP (16,6-15,1 ka BP) (Figure 1) (Prates et al., 2020), which also reinforces a pre-Clovis and post-LGM timeline for the earliest human settlement of South America, or at least for the intensification of this process.

Demographic models of population dispersion reveal that if the first inhabitants of Brazil arrived at 13,8 ka BP departing from the western opening of the Cordilleran glacier (i.e. Pacific coastal corridor) at 17 ka BP, the required dispersion rate would be 4,1 km per year, a value within the range of what is seen for present-day hunter-gatherers (Delser et al., 2021). In any case, the continent was already occupied mainly between 13,2-12 ka BP (Figure 1) (Sutter, 2021; Delser *et al.*, 2021), with the Isthmus of Panama serving as an entry point and the initial dispersal taking place along the Pacific coast (Wang et al., 2007; Bodner et al., 2012; Lindo et al., 2017; Braje et al., 2017; Brandini et al., 2018) and the Atlantic coast (Reich et al., 2012; Gómez-Carballa et al., 2018). However, any traces of this initial occupation that were left on the coastal continental shelves exposed during the LGM are now most likely under more than a hundred meters of water from the Pacific or Atlantic Oceans, severely limiting access to the vestiges of the initial settlement of the American continent as a whole, as well as biasing inferences about the date of arrival and the migratory routes used. This might, once again, skew the evidence in favor of a more recent human presence on the continent rather than a pre-LGM or peri-LGM timeline.

At first glance, the population history of South American natives also appears to inevitably reflect a post-LGM initial presence, because their ancestry can be traced back to the SNA lineage, albeit with a significant genetic structure formed most likely inside the Americas. However, a relative excess of genetic affinity with Australasian populations - including groups from Australia, Melanesia, and South Asia - was detected in some contemporary indigenous communities from the Amazon (Karitiana and Suruí) and the Brazilian central plateau (Xavante) (Skoglund et al., 2015). It was also found in an ancient individual from the Lagoa Santa site in Minas Gerais, with 10 ka BP (Figure 1) (Moreno-Mayar et al., 2018b). More recently it was found in at least two additional modern-day groups from the Central-West Brazilian region (Guaraní Kaiowá) and the northern Peruvian Pacific coast (Chotuna) (Figure 1) (Castro e Silva et al., 2021), revealing a much more widespread distribution of this ancestry contribution. This relative excess is expressed especially when some South American populations are compared to specific Mesoamerican populations such as those from the Mixe ethnic group, which are the descendants of one of the most deeply diverged SNA lineages and an outgroup to South American natives (Reich et al., 2012). It is also interesting to note that the Mixe exhibit a significant sign of contribution from an unsampled population - labeled unsampled population A (UPA) - which probably diverged during the period of standstill in Beringia from the ancestral population that presumably was already genetically structured (Moreno-Mayar *et al.*, 2018b). It is unknown whether the gene flow from UPA into Mixe interferes with the detection of this relative excess allele sharing; however, this is unlikely the case, as it was previously demonstrated that other groups, for which no evidence of this gene flow has been found, exhibit the same pattern as Mixe; *id est*, some Native American groups also present less shared genetic drift with Australians than other American indigenous communities (Castro e Silva *et al.*, 2021). 

This excess affinity with Australasians was modeled as the contribution of another unsampled population, the now-famous “Ypikuéra” (ancestral in Tupi languages) or “Y” population, which suggested a more complex population history than had been anticipated until that point, most likely involving an additional population influx from Beringia into the continent or the existence of a major genetic structure in Native Americans’ ancestors (Skoglund et al., 2015). In any case, the proportion of this extra ancestry in the groups where it was discovered is quite low, ranging from 1 to 3% of the total (Skoglund *et al.*, 2015, Moreno-Mayar et al., 2018b, Castro e Silva et al., 2021a).

Interestingly, it has been suggested by a recent study that the divergence between the AB, NNA, and SNA groups might have taken place in Asia (Ning et al., 2020), which would increase the probability of contact and gene flow from East Asian groups, including a possible gene flow from groups related to contemporary Australasian populations exclusively into the SNA branch. It should be highlighted, however, that this genetic affinity pattern is completely consistent with other scenarios in which gene flow from other Asian sources with common ancestors with present Australasians occurs.

Thereby, the existence of this Australasian signal opens up a myriad of possibilities for the initial peopling of the Americas, at least from a genetic standpoint. Most intriguing, this involves the possibility of a very early human presence on the continent during or even before the LGM, as long suggested by several archeological sites, though given the lack of pre-Clovis human skeletons, there is still significant debate among archeologists over whether the stone tools discovered were man-made or naturally occurring flakes, among other aspects regarding the validity of these peri and pre-LGM sites (Sutter, 2021). Nonetheless, the recent discovery of very solid archeological evidence of the human presence in the Chiquihuite Cave around at least 19 ka BP and the ancient footprints in New Mexico with 23 ka BP (Ardelean et al., 2020; Bennett et al., 2021) is contributing to what seems to be a final push in the direction of a new paradigm that humans were present in the Americas during or even before the LGM. 

Notably, this possibility of an additional population influx is not new and has previously been hypothesized based on the existence of the so-called “Paleoamerican” cranial morphology, which has been observed in some individuals from the Lapa do Santo site in Brazil as well as other regions such as Baja California in Mexico (Neves et al., 1996; Powell and Neves, 1999; González-José et al., 2005). In this two-component model, these Paleoamerican individuals would represent early settlers of the continent with distinct morphology and genetic ancestry in comparison to the later migrants which gave rise to contemporary Native Americans. Nonetheless, this model of two distinct ancestry components was challenged by craniofacial morphological analyses, which revealed extensive morphological diversity, implying that the Paleoamerican and Native American craniofacial morphologies would be only the extremes of the spectrum of variation, with the first preserving a higher proportion of ancestral characters that would have been more prevalent in the groups of the initial settlement during the Pleistocene, while the latter would present a larger set of derived phenotypes such as facial flattening, which would have evolved and dispersed from of the Arctic during the Holocene (González-José *et al.*, 2008; Bortolini et al., 2014). In any case, the hypothesis of an association between genetic and morphological diversity was tested in Native American individuals, and it was found that those individuals identified as having a Paleoamerican morphology do not show a significant excess of allele sharing with Australasians, with only one exception, and for this reason, such individuals could more parsimoniously be considered descendants of the same ancestral groups as the other Native Americans, both ancient and contemporary, without the requirement for any additional ancestry contributions (Posth et al., 2018; Moreno-Mayar et al., 2018b).

Taken together, the current archeological evidence supports that humans were present in the Americas at least 20 ka BP during the peak of the LGM (Ardelean et al., 2020; Bennett et al., 2021). Although the identity of these first Americans remains an open question, genetics has given us some insight into who they may have been, as revealed by the faint signal of shared ancestry with modern-day Australasian peoples (Skoglund et al., 2015; Castro e Silva et al., 2021). This data supports the hypothesis that the initial settlers were more closely related to the ancestors of modern Australasians than to those of East Asians and also implies that their contribution to post-LGM Native American populations was mostly absent and seldom minimal. In this scenario, these first human groups to reach the continent would have been later replaced by the ANA descendants, starting by the end of the LGM, and only rarely would have admixed with them. Furthermore, our recent findings show a lot of variation within populations (Castro e Silva *et al.*, 2021), which suggests that some of these first Americans could have lived in relative isolation until very recently when admixed with SNA populations. Furthermore, most of the genetic contribution from these early ancestors might have been erased by the intense population dynamics during the Holocene and by the successive inflows of distinct SNA groups in the case of South America (Posth et al., 2018). 

Considering that continental glaciers completely blocked northern North America throughout the LGM period, an early settlement of the Americas requires an alternate pathway. This alternative is provided by the so-called coastal migration theory (CMT) (Davis and Madsen, 2020), which proposes that the Pacific Rim shorelines were used as a route into the Americas from Asia by groups of humans adapted to a seaside lifestyle, likely based on the exploitation of the resourcefully rich environments of kelp forests present along both continents’ Pacific coasts (Erlandson et al., 2007). In that case, it could also help explain the absence of the Australasian signal in North America if the dispersal was rapid and mostly restricted along the Pacific coast, resulting in more significant population growth in South America at the expense of North America. Most interestingly new evidence points to the existence of a very large number of islands in the Bering Sea to the south of Beringia between 30 and 8 ka BP, named the Bering Transitory Archipelago (BTA), which would have greatly enhanced the availability of marine resources and also facilitated sea travel through more easily navigable and protected waters (Dobson et al., 2021).

Finally, some genetic and morphometric analyses of Northeastern and Southeastern Asians (NEA and SEA, respectively) provide some intriguing pieces of evidence on the origins of the Native American-Australasian connection. First, a link between the Onge and the ancient SEA hunter-gatherers, known as Hòabìnhians, is demonstrated by the genetic affinity between the Onge and two Hòabìnhians from Laos and Malaysia with approximately 8 and 4 ka BP (McColl et al., 2018), implying that the latter are closely related to the Onge’s ancestors. Second, morphological affinities between the Onge (a SEA population) and the NEA (Matsumura et al., 2019) support the hypothesis that a group closely linked to the Onge - thus also likely related to the Hòabìnhians - was involved in an admixture event with the ANA and thus responsible for the Australasian genetic affinity observed in indigenous Americans (Skoglund et al., 2015). There is also solid evidence that the distribution of modern-day Australasian and East Asian ancestors was significantly different across East Asia during the Pleistocene and that populations like the Jomon from Japan show very clear indications of a mixture of northern and southern Asian ancestries (McColl *et al.*, 2018; Wang et al., 2020); this is particularly meaningful given that Japan is a likely candidate and the proposed birthplace of the First Americans in the CMT framework. These findings illustrate how important it is to comprehensively elucidate East Asian population history in order to grasp the full picture of the Americas’ peopling.

## The South American roots of human diversity

In South America, current data suggests that at least three distinct SNA populations dispersed into the continent (Posth et al., 2018). The first would be representatives of an SNA group genetically close to the Anzick-1 (Rasmussen et al., 2014), while the second SNA influx, which lacked this particular affinity for Anzick-1, began to replace the first group by around 9 ka BP, indicating at least partial demic replacement (Figure 1) (Posth *et al.*, 2018). In addition to these two main dispersions, another contribution was also identified, in this case, for a more specific and geographically restricted set of populations. This third population influx is represented by a set SNA groups genetically related to ancient individuals from the California Channel Islands which likely replaced, or at least made large contributions to, the populations in the central portion of the Andes, spreading to the region before 4,2 ka BP (Figure 1) (Posth *et al.*, 2018); interestingly this population movement may be linked to the agriculture dispersion from Mesoamerica (Sutter, 2021). Therefore, the overwhelming majority of genetic and archeological evidence trace back to population events initiated with the end of the LGM (Posth *et al.*, 2018; Moreno-Mayar et al., 2018b). However, as previously discussed, the existence of Australasian ancestry affinity signals (Skoglund et al., 2015; Castro e Silva et al., 2021) and archeological sites dating from the LGM era or earlier - e.g. (Guidon, 1986; Vialou, 2003) - suggests that humans may have been on the continent for a far more extended period.

South America, just like North America, according to the available evidence, was settled by very rapid population dispersions along the coast, but this time it most likely occurred along both the Pacific and Atlantic coastlines (Wang et al., 2007; Bodner et al., 2012; Reich et al., 2012; Lindo et al., 2017; Braje et al., 2017; Brandini et al., 2018; Gómez-Carballa et al., 2018). In this way, the initial human populations in South America, which arrived around 16-15 ka BP (Bodner *et al.*, 2012; Rasmussen et al., 2015; Dillehay et al., 2017; Prates et al., 2020), likely separated very early into two groups that spread independently along the west and east coasts (Figure 1); while there was possibly some occasional interaction and gene flow between them (Bodner *et al.*, 2012; Gómez-Carballa *et al.*, 2018), the Andean cordillera (Fuselli et al., 2003; Reich *et al.*, 2012) and the Amazonian forest (Gómez-Carballa *et al.*, 2018) presumably acted as deterrents to gene flow and also influenced this pattern of genetic differentiation. 

Another hypothesis argues that the Andes, Amazon, and coastal areas were colonized by three separate lineages that split before entering South America (Rothhammer and Dillehay, 2009). Although the Andean area may have simply been settled by a secondary splitting of the Pacific coastal branch (Skoglund and Reich, 2016). Population history models, at least in Peru, support the latter hypothesis (Skoglund and Reich ,2016); however the inferred split date (roughly 12 ka BP) (Harris et al.. 2018) between the three primary regions - namely Pacific coast, Andes and Amazonia - is aligned with the trifurcation hypothesis (Rothhammer and Dillehay 2009) and also shows that these major splits occurred relatively early during the settlement of South America (Figure 1). This divergence time also overlaps with the earliest archaeological findings in Peru and in the Brazilian Amazon, dated between 11 and 12 ka BP (Scliar et al., 2014); revealing that people have been inhabiting and adapting to the Andean highlands and the Amazonian rainforest environments for a very long period. In this sense, archeological findings indicate that permanent settlements start to appear in the Andes circa 9 ka BP, while genetic analyses point to a long-standing genetic continuity in the Lake Titicaca region possibly from 3.8 or even 7 ka BP up until the present-day Aymara and Quechua speaking peoples of the same area (Lindo et al., 2018). Indeed, some level of long-term genetic continuity inside large continental areas - such as the Pacific coast, the Andes or the Amazon - seem to be common, although sometimes the genetic affinity patterns between ancient and contemporary individuals suggest the occurrence of large scale population movements, at least among adjacent regions (Castro e Silva et al., 2022).

Conversely, eastern South America present some of the earliest post-LGM human remains in the Americas, such as the Lagoa Santa and Lapa do Santo sites in southeastern Brazil, with 10.4 and 9.6 ka BP, respectively; with individuals from both sites inferred to be descendants of the first SNA population influx (Figure 1) (Posth et al., 2018; Moreno-Mayar et al., 2018b). As previously stated, beginning approximately 9 ka BP, a new SNA group of people started to arrive, at least partially displacing the early migrants, as seen by their reduced affinity with the Anzick1 and their higher affinity with modern South American indigenous peoples (Posth *et al.*, 2018; Moreno-Mayar *et al.*, 2018b). There appears to be a robust genetic affinity between Jê-speaking communities in central and southern Brazil and ancient individuals from across the area, especially those from the early Holocene (Castro e Silva et al., 2020; Castro e Silva *et al.*, 2022). Aside from that, Jê-speakers have an exclusive ancestry component, making them the most distinct group of eastern South Americans in terms of genetic structure, implying that they are descended from a different branch, possibly a more basal one, with higher genetic contributions from ancient populations from the region (Castro e Silva *et al.*, 2020; Castro e Silva *et al.*, 2022). 

It is worth noting that the Atlantic coast, as well as riverside and lake areas, have been occupied by multiple and likely diverse fisher-gatherer communities from at least 8 ka BP (Figure 1), which are especially known by the shell mounds they erected, known as Sambaqui, the term Tupi-speakers used to call them (Gaspar et al., 2008). Furthermore, there is a strong possibility of a much earlier presence on the shoreline that would have been completely erased or concealed by the Holocene’s rising sea levels. Hence, these groups would have occupied the Brazilian coast from at least 8 ka BP until the arrival of Tupi-Guarani groups and Macro-Jê speakers, not necessarily in that order, as evidenced by the presence of ceramics from the Tupiguarani and Taquara/Itararé traditions in the uppermost layers of some sambaqui, respectively (Gaspar *et al.*, 2008). The relationship between the Sambaqui mound builders and the contemporary indigenous communities remains largely unknown due to the relatively small number of individual analyzed until now, however current evidence suggests a higher affinity between them and present-day Jê-speakers, in comparison to other eastern South American natives (Castro e Silva et al., 2020; Castro e Silva *et al.*, 2022).

Regarding the Southern Cone, the earliest evidence of human activity dates back to 14.5 ka BP at the Monte Verde site in Patagonia (Dillehay, 2009), point to an extremely rapid settlement after the initial arrival in the continent at 16-15 ka BP (Bodner et al., 2012; Rasmussen et al., 2015; Dillehay *et al.*, 2017; Prates et al., 2020). Even Tierra del Fuego, the continent’s southernmost point, was populated prior to 8 ka BP, when it was still connected to South America due to much lower sea levels (Morello et al., 2012). Furthermore, despite some morphological diversity, as previously discussed, once interpreted as remains of early migrants with Paleoamerican skull morphology and a distinct ancestry (Neves et al., 1996; Powell and Neves, 1999; González-José et al., 2005), ancient and contemporary Patagonians are descended from the same northeastern Asian lineages as other indigenous Americans (i.e., SNA branch), and hence have no excess affinity with Australasians (Raghavan et al., 2015; Skoglund and Reich, 2016); beyond that, they present higher genetic affinities with each other and also with present-day indigenous communities in Central-Southern Chile (Raghavan *et al.*, 2015; de la Fuente *et al.*, 2018). 

Following initial population dispersals, South America’s diverse climates and environments resulted in a demographic and evolutionary history that varied greatly through time and space. According to the most comprehensive analysis of the spatial-temporal distribution of calibrated radiocarbon datings (with 5,464 datings from 1,147 archeological sites), ranging from 13 to 2 ka BP, this demographic history is divided into two main phases with distinct demographic dynamics (Goldberg et al., 2016). During the first phase, between 13 and 5.5 ka BP, there was an initial rapid geographic expansion with the occupation of much of the continent, followed by a stage of density-dependent population growth, so that at first the population increased rapidly until the carrying capacity was reached and from that point onwards population sizes remained relatively constant between 9 and 5.5 ka BP. This early stage of logistic growth is further supported by a recent analysis of a high-quality curated collection of radiocarbon dates from the early settlement era (Prates et al., 2020), which reveals that demographic stability was actually reached by 11 ka BP. 

Whereas, the spread of sedentary lifestyle and intensification of food production consolidating around 5.5 ka BP, initiated a new period of exponential population growth, at least in some cultural centers, particularly those located in the central and northern Andes. According to this model, more than half of the population growth occurred during this second stage (Goldberg et al., 2016), although other studies have found that population growth rates varied greatly between regions and through time, pointing to a significantly earlier expansion in the Andes, beginning about 9 ka BP, in comparison to other places in the east, such as Patagonia, where a more gradual and late expansion begins between 7.5 and 5 ka BP (Perez et al., 2016; Perez *et al.*, 2017; Prates et al., 2020).

This shift in human population growth rates also overlaps with a change in the climatic pattern that happened throughout the Middle Holocene, where the climate that was formerly dry and variable in precipitation entered a phase of consistent precipitation increase in the Southern Hemisphere’s tropical forests (Iriarte et al., 2017). If, on the one hand, the Middle Holocene’s driest period coincided with a population decrease (beginning at 8.6 ka BP) (Riris and Arroyo-Kalin, 2019), on the other hand, this increase in precipitation led to an expansion of tropical rainforests between 5 and 1 ka BP and increased human population growth and movement, particularly in the southern Amazon forest (Iriarte *et al.*, 2017). 

This transition to wetter climates is also associated with an increase in sedentism as well as in the prominence of agriculture as a subsistence strategy (Goldberg et al., 2016), as indicated by the increased frequency of landscape modifications during the late Holocene (Iriarte et al., 2020). In turn, the onset of plant domestication in South America broadly overlaps with the extinction of the last megafauna species about 9-8 ka BP (Borrero, 2009; Martínez et al., 2016), with certain plant species becoming totally domesticated by 6 ka BP (Larson et al., 2014), although the first use of various plants occurred yet in the Late Pleistocene or Early Holocene (Iriarte *et al.*, 2020). The resulting boost in food production did not directly translate into an increased rate of population growth; rather, this occurred mainly in a few cultural centers - especially in the Andes - where intensive agricultural systems assumed precedence as the primary subsistence strategy (Goldberg *et al.*, 2016; Perez et al., 2017; Sutter, 2021). 

Some studies suggest that this exponential rate of growth was maintained until the arrival of Europeans, at least in Amazonia (Arroyo-Kalin, 2018), while others indicate a slowdown in growth or even a decrease in population size in some areas, possibly due to having reached carrying capacity, autochthonous diseases, or even climate and social change (Arroyo-Kalin and Riris, 2021; Bush et al., 2021). However, as expected, the majority of genetic and archeological data pointed to the highest mortality rate occurring after Europeans arrived in the Americas, probably peaking later in the colonization period (Browning et al., 2018; Jones et al., 2021; Castro e Silva et al., 2022).

In addition, the contrast between western and eastern population growth regimes, among other factors, prompted the development of a useful model known as the Andes-Amazon divide (Pearce et al., 2020), which was applied for a long time to understand the geographical distribution of many archeological, ethnolinguistic, genetic, and demographic patterns; however, it limited and biased the way South American indigenous peoples were studied, particularly in relation to the genetic diversity (Barbieri, 2020; Fehren-​Schmitz, 2020; Santos, 2020). According to this perspective, an evolutionary model was proposed in which opposing dynamics of evolutionary forces were historically at work in the Andes and Amazonia (Tarazona-Santos et al., 2001; Santos, 2020). In essence, the Andes would have been occupied for a long time by large populations with evermore intensive food production systems, eventually giving rise to highly hierarchical and interconnected societies with very similar environmental and cultural conditions; given the large population sizes and widespread gene flow, this supported the preservation of increased genetic diversity within populations while decreasing genetic differentiation across populations. Conversely, the Amazonia would have been inhabited by small and isolated predominantly hunter-gatherer groups living in very heterogeneous environments with substantial interpopulational cultural differentiation, resulting in low within population genetic diversity and high among population genetic differentiation, due to the low gene flow. 

However, as evidence of denser occupation and the presence of more complex cultures in the Amazon grows, these discrepancies in cultural and demographic complexity between Andes and Amazonia are being reassessed (Heckenberger et al., 2003; Heckenberger and Neves, 2009; Roosevelt, 2013; Piperno et al., 2015; Clement et al., 2015; Pearce et al., 2020). Indeed, existing evidence indicates that highly populated permanent settlements existed along major rivers (Piperno *et al.*, 2015), and also that Amazonia was a major world center of crop domestication, with at least 83 species having been domesticated to some extent (Clement *et al.,* 2015). These processes were also associated with extensive environmental modifications, such as the formation of domesticated landscapes, exemplified by the Amazonian dark earths (ADEs), which first appeared around 6 ka BP during the mid-Holocene, and became widely distributed by 2.5 ka BP, and were crucial for both plant domestication and food production, and thus for the increased population growth rates (Clement *et al.*, 2015; Neves and Heckenberger, 2019; Iriarte et al., 2020). 

The development of landscape and crop domestication are different types of niche construction, which is perhaps the most permanent and evident manifestation of a long process of human adaptation that, differently from the general evolutionary process, shifts environmental selective pressures in favor of both humans and their domesticated (or semi-domesticated) species and leads to coevolutionary dynamics between human genes and culture (Kendal et al., 2011; Hünemeier et al., 2012a; O’Brien and Laland, 2012; Flores and Levis, 2021). In the case of Amazonia, this evolved through interactions between human groups and the extremely diverse Amazonian environments, mediated by both biological and cultural evolution, and resulting in one of the world’s most culturally diverse areas as well as in intensive and widespread landscape modifications (Roosevelt, 2013; Piperno et al., 2015; Clement et al., 2015; Pearce et al., 2020).

Despite the enormous environmental and cultural diversity of South America and Amazonia, there is no equivalency in terms of genetic diversity levels, which are exceedingly low when compared to populations on other continents (Bergström et al., 2020; Castro e Silva et al., 2022). This low genetic diversity is manifested as a low level of heterozygosity, which decreases in a gradient that begins in the north of North America and goes until southern South America, whereas in South America a second gradient is directed from west to east (Wang et al., 2007; Reich et al., 2012; Castro e Silva *et al.*, 2022), both likely tracing back to the initial population events and the serial population bottlenecks faced by these groups, because the effect of genetic drift in small and isolated groups is stronger, resulting in an increased rate of genetic diversity loss; for this reason the Amazon may be home to the world’s living populations with the lowest levels of genetic diversity (Bergström *et al.*, 2020; Castro e Silva *et al.*, 2022). Furthermore, isolation by distance also likely plays a significant role in shaping these gradients of genetic variation (Castro e Silva *et al.*, 2022). Concurrently, genetic divergence among groups - measured by statistics such as Fst - tends to increase from north to south in the Americas and from west to east in South America for the same reasons (Wang *et al.*, 2007; Reich *et al.*, 2012; Castro e Silva *et al.*, 2022).

### The spread of genes and culture in the Late Holocene

As in other regions of the world, crop and landscape domestication, sedentarization, and intensification of food production led to higher rates of population growth in some cultural centers of South America, which in turn caused an increase in human population movement (Loog et al., 2017; Delser et al., 2021). This process eventually resulted in demic expansion events, which were responsible for significantly restructuring the landscape of genetic and cultural diversity, specially during the Late Holocene period, not only in South America, but also globally (Sokal et al., 1991; Cordaux et al., 2004; Wen et al., 2004; de Filippo et al., 2012; Ammerman and Cavalli-Sforza, 2014). 

Continental-scale genetic studies of American indigenous populations have not found any significant relationship between genetic and cultural diversity (Hunley et al., 2007; Roewer et al., 2013; Bisso-Machado and Fagundes, 2021), although a large sample of South American indigenous groups showed at least partial correlation between autosomal genetic variation and ethnolinguistic diversity (Castro e Silva et al., 2022). Furthermore, it is also possible to find unambiguous examples where culture has had a considerable impact on genetic patterns, particularly in more local contexts, such as the Xávante of Brazil’s central plateau, who were impacted by fission-fusion population dynamics in which populations split and migrate in a non-random manner motivated by cultural factors, later evolving independently or merging back together, or even merging with other groups from the same ethnicity (Neel and Salzano, 1967). This cultural trait, along with the fact that the Xávante groups are highly endogamous, led to an acceleration in the phenotypic differentiation when compared to other populations (including its genetic and linguistic sister group, the Kayapó), as demonstrated by the rapid evolution of their craniofacial morphology (Hünemeier et al., 2012b).

Moreover, cultural variables relating to subsistence strategies are likely to be the primary determinants affecting the broader patterns of genetic structure, particularly after the enormous population dispersals of agriculturalist and herder peoples during the Holocene (Sokal et al., 1991; Cordaux et al., 2004; Wen et al., 2004; de Filippo et al., 2012; Ammerman and Cavalli-Sforza, 2014). Thereby, for instance, the relatively homogeneous genetic landscape of Andean peoples could be the outcome not only of higher rates of gene flow, as previously mentioned (Tarazona-Santos et al., 2001; Santos, 2020), but also of past demic expansion events of agriculturalist populations (Barbieri et al., 2017; Barbieri *et al.*, 2019) inferred to have originated on the Pacific coast (Stanish, 2001), in such way that even today, groups inhabiting the central Andes speaking Uro languages and descended from hunter-gatherers are genetically differentiated from agriculturalists who speak Aymara and Quechua (Sandoval et al., 2013). 

Eastern South America is no exception, as populations with a predominance of agriculturalist subsistence strategies expanded out of the Amazon. In this sense, Tupi-speaking groups present a pattern of isolation by distance consistent with a past population expansion, in line with the expectations of the Tupi Expansion hypothesis, whereas groups speaking Jê languages and primarily hunter-gatherers show a non-linear pattern of dispersion, which contradicts the expectation of past demic expansions (Noelli, 2008; Ramallo et al., 2013). Although it is important to note that pre-Columbian Amazonian peoples (i.e., Tupi-speakers) developed a very distinct type of food production system known as polyculture agroforestry, which combined the cultivation of domesticated plants with the management of semi-domesticated ones amidst forest environments; which is a very different strategy from that of other agriculturalists worldwide, that in general involved an emphasis on monoculture of one or a few cereal species in homogeneous environments (Neves, 2013; Gregorio de Souza et al., 2020; Iriarte et al., 2020). The distinctiveness of the polyculture agroforestry strategy may also help explain why the impact of indigenous Amazonians was smaller than expected for the inferred population sizes (Piperno et al., 2021; Castro e Silva et al., 2022).

Some of South America’s largest language families originated and spread from the Amazon. Furthermore, an intriguing relationship has long been proposed between the geographic distribution of some language families and some of the most important Late Holocene material culture traditions (Dixon et al., 1999; Neves, 2011); while there is no exact correspondence between them, current evidence supports the hypothesis that at least some of these languages and traditions were dispersed together through demic diffusion (Noelli, 2008; Castro e Silva et al., 2020; Gregorio de Souza et al., 2020; Nägele et al., 2020). In this sense, it is hypothesized (Gregorio de Souza *et al.*, 2020) that the four largest linguistic families in South America, namely Arawak, Karib, Jê (part of the Macro-Jê stock) and Tupi-Guarani (part of the Tupi stock) are respectively related to the traditions Saladoid-Barrancoid (Lathrap, 1970; Brochado, 1984), Incised-Punctate (Lathrap, 1970), Una (Brochado, 1984; Noelli, 2008) and Tupiguarani (Noelli, 2008; Corrêa, 2014). Among these traditions, only Saladoid-Barrancoid and Tupiguarani extended beyond the Amazonian basin; the former reached the islands of Puerto Rico and Hispaniola in the Caribbean (Keegan, 1995), while the latter spread over 5,000 kilometers across a vast expanse of eastern South America, including the central Brazilian highlands, the Caatinga in northeastern Brazil, the Atlantic forests of southern and southeastern Brazil, and the Argentine pampas (Noelli, 1998; Noelli, 2008). The Tupiguarani tradition itself is subdivided into three major subtraditions: Guaraní, Amazon Tupinambá, and Atlantic Forest Tupinambá, which predominantly occur in the Paraná Basin, southeastern Amazonia and Atlantic coast, respectively (Almeida and Neves, 2015). 

Conversely, the languages of the Arawak and Tupi-Guarani families extended over the territories where their respective material culture traditions thrived and were spoken by local peoples until the European conquest (Davis and Goodwin, 1990; Urban, 1992; Rodrigues and Cabral, 2012). Indeed, for a long time, linguistic and cultural similarities between the Guaraní and the Tupi were observed, allowing their unification into a single group known as the Tupi-Guarani; so as a consequence, the Tupi-Guarani is the linguistic family with the widest distribution in Brazilian territory, integrating the Tupi stock with 9 other families restricted to the Amazon basin (Urban, 1992; Rodrigues and Cabral, 2012).

The Tupi-Guarani family and the Tupi expansion are quite emblematic and revealing of the implications of Late Holocene demic expansions to the South American genetic landscape. Currently it is known that all families of the Tupi stock had a common center of origin, most likely located in southwestern Amazonia (Noelli, 2008), between the Madeira and Guaporé rivers, the so-called Madeira-Guapore region, which contains the highest linguistic and genetic diversity of Tupi-speakers (Walker et al., 2012; Ramallo et al., 2013; Santos et al., 2015). Furthermore, the association between field archeology data, Carbon-14 date distribution, historical linguistics, and ethnohistorical sources also supports this location as their center of origin (Miller, 2009). The Tupi-Guarani homeland was most likely located in southeastern Amazonia between the Xingu and Tocantins rivers, where their greatest linguistic and material culture diversity exists (Figure 1) (Almeida and Neves, 2015). The Tupi-Guarani expansion would have begun about 2.4 ka BP and would have reached the Paraná basin around 2.2 ka BP and the Atlantic coast by at least 1.8 ka BP (Figure 1) (Noelli, 2008; Macario et al., 2009). 

Current data, including genetic analyses, supports a demic diffusion model along the lines of a long-standing hypothesis that the Tupi-Guarani spread out of Amazonia as a result of ongoing population expansion caused by the emergence of their agriculturalist food production systems (Brochado and Lathrap, 1982; Brochado, 1984; Noelli, 2008; Castro e Silva et al., 2020; Corrêa,, 2014). In this context, one branch of the Tupi-Guarani speakers headed southwards and into the Paraná basin, giving rise to the Guaraní, and another branch moved towards the mouth of the Amazon river and then dispersed along the Atlantic coast down to the present-day Southeastern Brazil (Figure 1), these latter were the ancestors of the coastal Tupi-Guarani - sometimes referred to as Tupinambá. Furthermore, paleoecological and paleoclimatic findings (Iriarte et al., 2017) implies that a wetter climate in the Late Holocene drove forest expansions in the southern Hemisphere between 3 and 2 ka BP, during the Tupi Expansion’s beginning. The expansion of riverine forests, in particular, would have created an ecological opportunity for the Tupi-Guarani to expand, by providing the necessary environmental conditions for food production through the polyculture agroforestry to which they were adapted, and possibly also contributing to the group’s expansive ethos.

### The current picture of South American indigenous ancestries

Humans initially arrived in the Americas during or even before the LGM, however the genetic diversity of Native and non-Native American peoples has been greatly altered by the recent events triggered by European arrival. The European colonization of this continent triggered some of the greatest demographic and migration events in human history. At the time of arrival, tens of millions of Native Americans were living on the continent (Thornton, 1987; Denevan, 1992). As widely known this massive population contingent was drastically reduced, by approximately 90-95%, from 1492 onwards; as a consequence of different processes arising from the European colonization, such as epidemics, enslavement, instigation of violence between rival indigenous groups, wars of conquest, forced displacement of territories, habitat destruction, disruption of subsistence strategies and traditional knowledge (Thornton, 1987; Stannard, 1993). 

Concomitantly, there was also widespread miscegenation between these peoples, previously separated by thousands of years of evolutionary history and which now met on the American continent (Adhikari et al., 2016, 2017; Ongaro et al., 2019). Thus the ancestry of contemporary Latin American populations is predominantly tripartite, tracing back their origins to the indigenous American ancestors, the European colonizers and the enslaved Africans forcibly brought to the Americas during the Transatlantic Slave Trade (Adhikari *et al.*, 2016, 2017; Ongaro *et al.*, 2019).

This process of admixture occurred in a differential manner throughout time and space, heavily influenced by the local contexts of indigenous population density, availability of specific resources of interest to Europeans, and the consequent volume of immigrants; but also dependent on other factors such as the intensity of African and indigenous slave labor employment in each specific region, as well as the social and cultural contexts that determined the frequency and volume of immigration and admixture (Adhikari et al., 2017). Furthermore, considerable macro and micro regional population migrations within the continent have occurred over time, leading the distribution of this mosaic of ancestries to shift even more until reaching the current configuration (Ruiz-Linares et al., 2014; Montinaro et al., 2015; Adhikari *et al.*, 2016; Chacón-Duque et al., 2018; Ongaro et al., 2019). Admixture involving indigenous peoples, in particular, occurred preferentially locally, so that today the indigenous ancestry of contemporary admixed populations recapitulate consistently to groups that occupied the same region in the past, in such a way that the study of these populations is able to reveal the pattern of structure and genetic diversity from the pre-contact period (Harris et al., 2018; Gnecchi-Ruscone et al., 2019; Barbieri et al., 2019; Castro e Silva et al., 2020; Castro e Silva *et al.*, 2022).

Notably, the process of European colonization appears to have had a less severe impact on the indigenous people of the Andes, with slightly smaller population declines and admixture (Harris et al., 2018; Gnecchi-Ruscone et al., 2019; Barbieri et al., 2019; Castro e Silva et al., 2022). On one hand, existing data indicates that the Amazon also acted as a refugium, at least in terms of avoiding extensive admixture; on the other hand, these Amazonian populations experienced significant population reductions, in the same way as other South American populations, as demonstrated by inferences of historical effective population sizes (Castro e Silva *et al.*, 2022).

Recent research has produced a more detailed picture of the genetic structure of South American indigenous peoples, demonstrating a pattern of genetic-geographic relationship (Gnecchi-Ruscone et al., 2019; Barbieri et al., 2019; Castro e Silva et al., 2022). First in western South America there are at least three major groups: (i) southern Andes (southern Peru), (ii) northern Andes (Ecuador and Colombia), and (iii) central Andes (northern Peru) and Pacific coast (Gnecchi-Ruscone *et al.*, 2019; Barbieri *et al.*, 2019; Castro e Silva *et al.*, 2022). In its turn eastern South America also presents a minimum of three clusters of genetic similarity, namely: (i) the Guaraní communities in southern Brazil, (ii) the Jê-speakers in the central Brazilian plateau and southeastern Amazonia, and (iii) the Tupi and Karib-speaking populations from the Amazonia (Castro e Silva *et al.*, 2022). Finally, western Amazonians were most likely formed through gene flow from Andean populations into the lowlands (Barbieri *et al.*, 2014; Harris et al., 2018; Gnecchi-Ruscone *et al.*, 2019; Barbieri *et al.*, 2019), and so appear as transitional populations, evidencing the absence of a hard genetic divide between the Andes and the Amazonia (Castro e Silva *et al.*, 2022).

Furthermore, indigenous communities in the Southern Cone are genetically distinct from other South American populations, to the point that Central-Southern Chile and Patagonia have an almost unique genetic component, which also has a long-term continuity in the region (de la Fuente et al., 2018). Furthermore, the Yámana, residents of the continent’s southernmost regions, also have a distinct genetic component, most likely a result of their history of isolation; in fact, the genetic diversity of Patagonians is mostly consistent with a demographic history of small and isolated populations (de la Fuente *et al.*, 2018). 

In general this broad scale population structure of South America was largely in place by the onset of the Late Holocene, resulting in patterns of long-standing genetic continuity (Willerslev and Meltzer, 2021; Castro e Silva et al., 2022), specially in the case of the Andes and Patagonia (de la Fuente et al., 2018; Harris et al., 2018; Lindo et al., 2018; Nakatsuka et al., 2020). Although, as previously discussed, South America likely was the stage for at least a few episodes of increased population growth and demic diffusion during the Late Holocene, which reshaped the significantly genetic landscape and appear to be predominantly linked to the intensification of sedentism and food production systems, as well as with a transition to wetter climates in the period (Goldberg et al., 2016; Iriarte et al., 2017; Neves and Heckenberger, 2019; Iriarte *et al.*, 2020). The rise of Andean empires most likely involved population increase in cultural centers, yet geographical expansion of these empires did not necessarily require population dispersals; indeed genetic continuity has been documented, in certain cases dating back to people from the mid Holocene (Nakatsuka *et al.*, 2020). 

The Amazon is the other major center of origin of population expansions in South America, where numerous indigenous communities were able to flourish despite quite different subsistence strategies and environmental challenges, and eventually some of them experienced substantial population growth and dispersed throughout the continent. The Arawak and Tupi expansions were most likely the biggest in terms of scope and impact, and their genetic imprint may still be seen in indigenous populations in eastern South America and the Caribbean today (Castro e Silva et al., 2020; Nägele *et al*., 2020).

In conclusion, research on native and admixed American populations has gradually revealed the enormous diversity of ancestral lineages for which the Americas represented a meeting point, some of which were separated by tens of thousands of years, such as European and African, brought with colonization and the Atlantic slave trade, respectively. Other lineages, such as those that split during the earliest settlement of Northeast Asia, Siberia, Beringia, and their journey to the Americas, were separated for a shorter period of time, but were nevertheless separated thousands of years ago, towards the end of the Pleistocene. In addition other ancient populations, not sampled thus far, contributed lineages that likely emerged earlier, such as the Australasian lineages, which presumably diverged during the settlement of Southeast Asia. Finally, population dynamics and dispersions, particularly in the late Holocene, caused by climate change as well as the gradual transition to sedentism and more intensive agricultural food production, significantly reconfigured the patterns of indigenous American genetic diversity through inter and intra continental population movements.
